# Assessing policy dialogues and the role of context: Liberian case study before and during the Ebola outbreak

**DOI:** 10.1186/s12913-016-1454-y

**Published:** 2016-07-18

**Authors:** Juliet Nabyonga-Orem, Mesfin Gebrikidane, Aziza Mwisongo

**Affiliations:** 1 Health Systems and Services Cluster, World Health Organization Regional Office for Africa, B.P. 06, Brazzaville, Congo; 2 Health Systems and Services, World Health Organization Liberia Country Office, PO Box 316, Monrovia, Liberia

**Keywords:** Policy dialogue, Ebola viral disease, Health system resilience

## Abstract

**Background:**

In the last decade participatory approaches have gained prominence in policy-making, becoming the focus of good policy-making processes. Policy dialogue is recognised as an important aspect of policy-making among several interactive and innovative policy-making models applied in different contexts and sectors. Recently there has been emphasis on the quality of policy dialogue in terms of how it should be conducted to attain participation and inclusiveness. However, there is paucity of evidence on how the context influences policy dialogue, particularly participation of stakeholders. Liberia’s context, which is characterised as post-war, highly donor dependent and in recovery from the recent catastrophic Ebola outbreak, provides an opportunity to understand the influence of context on policy dialogue.

**Methods:**

This was an exploratory study using qualitative methods. Key informant interviews were conducted using an interview guide. A total of 16 interviews were conducted, 12 at the national level and 4 at the sub national level. Data were analysed using inductive thematic content analysis.

**Results:**

The respondents felt that the dialogues were a success and involved important stakeholders; however, there were concerns about the improper methodology and facilitation used to conduct them. Opinions among the respondents about the process of generating and selecting the themes for the dialogues were extremely divergent. Both before and during the Ebola outbreak, the context was instrumental in shaping the dialogues according to the issue of focus, requirements for participation and the decisions to be made. Policy dialogues have become a platform for policy discussions and decisions in Liberia. It is a process that is well recognised and appreciated and is highly attributed to the success of the negotiations during the Ebola outbreak.

**Conclusions:**

To sustain and strengthen policy dialogues in future, there needs to be proper information sharing through diverse forums and avenues, stakeholders’ empowerment and competent facilitation. These will ensure that the process is credible and legitimate.

## Background

In the last decade participatory approaches to policy-making have gained prominence, becoming the focus of good policy-making processes [1–3]. Among several interactive and innovative policy-making models, policy dialogue is acknowledged and regarded as applicable in various contexts and different sectors [4]. In recent years policy dialogue has also featured in the health sector policy-making arenas. This move has been prompted by lessons from poor policy implementation emanating from top-down approaches with inherently limited participation of actors [5–7]. Policy dialogue also has been shown to facilitate consensus building on challenges, goals, interests and solutions through its participatory and engaging manner [8]. This can lead to effective joint planning and implementation of programmes. In this article we define policy dialogue as a dialogue that is central to policy and decision-making processes, and contributes to realising evidence based and consensual policy changes and decisions [3].

However, literature stresses that policy dialogue is only as good as the quality of the process [2, 8]. There is emphasis on the importance of how policy dialogue is conducted in terms of whether it is participatory and evidence informed, and if it has clear processes and methodologies, good facilitation and an overall credibility [1, 2]. Literature identifies essential elements of effective policy dialogues as follows [1, 3, 6]:

- The extent to which it is clear what is to be achieved through the dialogue, or clarity of intent;
- The balance of power, knowledge and ownership, or negotiating capital;
- The capabilities and characteristics of the actors or the individuals involved;
- The forum used for the dialogue, that is the formal and informal spaces and opportunities to understand each other’s values and interests;
- The evidence, which is the extent to which data and analysis inform the dialogue, and who the owner of the data is.

Scholars emphasise the role of context in influencing the elements mentioned above [9, 10] making it suitable for our study. Studies on policy dialogue in health such as those by Hinchcliff et al. [11] and Gyapong [12] show context to be an important factor in policy dialogue since it shapes collaboration and contributions of participants and stakeholders, their opinions, perceptions and decisions. However, there is paucity of literature on how context affects stakeholders’ engagements in policy dialogue, which would help to better understand the intricacies of the process for learning and application in future dialogues.

Liberia is one of the countries implementing the policy dialogue programme supported by the European Union, the World Health Organization (WHO) and Luxembourg. In 2011 these three entities entered into a collaborative agreement to support policy dialogue on national health policies, strategies and plans. Referred to as the EU-WHO Policy Dialogue Programme, this collaboration had the ultimate aim to improve health sector outcomes in the targeted countries, with an overall focus on the promotion of universal health coverage, people-centred health care and inclusion of health in all policies. More specifically, the programme aimed to build the capacities of participating countries to develop, negotiate and implement evidence-based and all-inclusive national health policies, strategies and plans and to monitor and evaluate them. The programme also works to strengthen country processes and aid effectiveness, where appropriate, in line with the principles of the International Health Partnership (IHP+). Liberia’s context, which is characterised as post-war, highly donor dependent and emerging from the recent catastrophe of the Ebola outbreak, provides a good opportunity to understand the influence of context on policy dialogues.

## Methods

### Context

Liberia is a post-civil war state, where the conflict, which ran between 1989 and 2002, devastated the country’s economy, human capacity, infrastructure and security [13]. Recovery efforts began in 2006 after President Johnson Sirleaf came to power [14, 15]. Despite the efforts and strides, Liberia still has a long way to go. It is estimated that it is one of the poorest countries in Africa, with an average per capita income of US$ 160 [16]. Likewise, the health indicators have suffered from the country’s plight. In 2010 life expectancy at birth was 59.3 and infant mortality rate per 1,000 live births was 91.3. In 2008 the maternal mortality rate per 1000,000 live births was 990 [14]. Several donors have come to assist Liberia rebuilt. However, the aid architecture has been rife with criticism, including its effect on harmonisation and alignment to Liberia’s needs [13, 15]. Recently, the Ebola outbreak weakened the already stressed systems, economy and performance, adding to the country’s predicaments [17]. There is one promising element, though: policy dialogues have been going on in the health sector to help strengthen policy-making. The main areas of focus have been the development, implementation and monitoring of the health sector strategic plan; integrated monitoring and evaluation; capacity building with a focus on planning and budgeting; and operationalization of health financing.

### Specific methods

This was an exploratory study using qualitative methods. The research was conducted primarily through key informant interviews with various stakeholders. The interviews were conducted using a guide and were carried out by a Liberian researcher conversant with qualitative research and the context. The interview guide was pretested with health systems technical officers in the WHO Regional office and adjusted accordingly prior to conducting interviews. The initial list of respondents was drawn with the officials responsible for the dialogues at the Ministry of Health (MoH). This was followed with snowballing to identify additional respondents until descriptive saturation [18]. Respondents included a range of stakeholders at the national level such as the MoH and other relevant ministries, donors and nongovernmental organisations (NGOs). The respondents at the subnational level included sub-national health management team members and NGO representatives. A total of 16 interviews were conducted with 12 national and 4 subnational level respondents. Table 1 presents details of the respondents and their organisations. Data were collected between June and August 2015 after the Ebola outbreak of 2014.

**Table 1:** Respondents’ organisations

Organisation	Number of respondents
National level	
Government	4
International partners	4
Nongovernmental organisations/civil society organisations	4
Sub total	12
County level	
Grand Bassa	2
Bong	2
Sub total	4
Grand total	16

### Study sites

The national level stakeholders were interviewed in Monrovia, the capital city and where their various institutions were headquartered. Grand Bassa and Bong counties hosted the county level interviews. These counties were selected based on ease and convenience of access in consideration of the resource and time limitations and travel constraints due to the heavy rains and poor road infrastructure.

### Data collection domains

Data collection focused on five broad areas and aimed to assess the effectiveness of the policy dialogue programme in Liberia. These areas were contextualisation and understanding of the dialogue, governance and management of the dialogues, policy dialogue processes, policy dialogue outcomes, and policy dialogues around the Ebola response. Table 2 lists the areas and parameters that were assessed.

**Table 2:** Data collection domains

Key Issue	• Parameters for assessment
Contextualisation and understanding of the dialogue	• Policy context • Understanding of the dialogue • Importance of the dialogue • Issues for national and sub-national level dialogues
Governance and management of the dialogue	• Governance and management structure and approaches
Policy dialogue processes	• Selection of dialogue themes • Clarity of the issue to be deliberated upon • Selection of dialogue speakers and participants to the dialogues • Presentations informed by evidence • Facilitation of the processes • Feedback mechanisms (reporting) and follow-up of recommendations
Policy dialogue outcomes	• Level of harmonisation of activities of partners • Degree of stakeholders’ alignment to one plan • Level of collaboration between national and sub-national levels • Health systems and health outcomes attributable to the dialogue process
Policy dialogue around the Ebola response	• Governance and oversight issues being addressed prior to the Ebola outbreak • Pre-Ebola experiences: level of coordination of existing structure, management vs. accountability, successes vs. challenges • Changes in the nature of the policy dialogues occasioned by Ebola (national and sub-national) • Changes in the dialogue processes occasioned by Ebola (national and sub-national)

### Data analysis

The transcripts, which were in English, were analysed by two coders both of whom are authors of this paper. As a first step to formal analysis, we read the interviews in detail looking for emerging issues in line with the study objectives and these were coded and categorised into themes. One of the coders created a start list of the themes that were in line with the objectives of the study. Both coders read through all the transcripts independently and coded them using QSR NVivo 10. The two coders then selected the quotations that best represented each theme and sub-theme. The quotations selected by both coders were included as representative of the others.

## Results

### Understanding and perspectives of stakeholders on the intent of the policy dialogue

In general there were various perceptions and different understanding of the intent of the dialogues. Some of the respondents perceived the dialogues to be part of a programme meant to find ways to supplement the shortfalls in funding in the form of unrestricted funds. Some assumed that the dialogues were the yearly meetings held by the MoH to review the health systems’ performance. Yet to others the dialogues were helpful in facilitating the health system to generate new ideas on the structure, processes, capacity and facilitation of its support systems. It was felt that the good intentions of the dialogues had been challenged by their limited funding. A number of respondents felt that the funding was very thin in comparison with the volume of activities proposed during the dialogues. One respondent noted,

*The policy dialogue funding allocation is spread thinly over many activities. And for some of the activities, in particular the review of the M&E policy and plan itself, I think we had over a 3-year period $10,000 per annum. But the first review spent in excess of that $10,000 annual allocation.* (National level stakeholder)

In addition, the respondents indicated that the disbursement of funds was not carried out according to the agreed plan.

The respondents’ views on the intent of the dialogues could be grouped under three main categories: increasing participation during policy-making, harmonising and aligning partners’ and government goals, and improving implementation of programmes.

### Participation in the dialogues and characteristics of the actors

Most of the respondents felt that to generate innovative ideas and best practices, the policy dialogues had to have good planning, management, participation and governance. There was general consensus that participation in the dialogues was good, and participation of different and important actors was highlighted on several occasions as a strong feature of the dialogues. The inclusion of both national and county level stakeholders also was lauded.

Some of the respondents felt that participation consistency was affected by other activities that the stakeholders had to attend and the involvement of top level officials at both the county and national levels, who often had proxies representing them. The quotation below demonstrates this,

*I think there is good representation of partners and government. Maybe the only issue is that the level of participation is not consistent sometimes when individuals who regularly participate and understand the issues send proxies who are not at their level.* (National level stakeholder)

The presence of a range of stakeholders with different capacities, ranging from top-level and international level experts to sub-national (county) level actors created a sense of insecurity for the lower level actors. This was exhibited when junior officials were confronted with questions during their presentations, as one respondent remarked,

*Some county health officers will just come and give information and figures and sometimes if people push them against the wall and ask lots of questions, they are not able to defend some of these things.* (County health representative)

The method employed in group work and discussions also was regarded as ineffective as it perpetuated the tendency for domination by a few powerful participants. In those circumstances the influential actors were able to drive their personal agendas, as a respondent noted,

*In group work people come and spend a couple of hours before they have something to say. I think sometimes it’s driven by just the fact that they need to get per diem. I agree that some of the stakeholders or participants have genuine interest but not all of them, so those who were vocal and who want to be heard will dominate the conversation or group work with their ideas, and those who were kind of shy will never be heard.* (National level respondent)

The time allocated for the dialogues was thought to be inadequate from a participation perspective. Cultural and societal contexts in Liberia commonly allow anyone to voice his or her opinion regardless of its sense. This was responsible for some prolonged, meaningless discussions in the dialogues at the expense of quality and relevant input.

There was general consensus that community participation was weak. The respondents expressed concern over the limited participation of the general community, which was contrary to the ministry’s declaration of the importance of community engagement. There was debate also on the definition of community participation, which was deemed ambiguous. For example, in one of the meetings the participation of superintendents and their empowerment to make decisions was considered as community participation, while in other contexts it was the community members, who were beneficiaries of the health services, who were actively involved in the dialogues.

The participation of the community was critical and genuine during the Ebola outbreak, as many of the respondents stated. This argument was based on the community members’ involvement in the dialogues during the outbreak. It was stressed that community participation was key in winning the battle against the Ebola virus disease. Through the dialogues government officials became aware of the societal constructs and myths surrounding Ebola and were able to counteract them.

### Characteristics and management of the dialogue forums

The organisation of the dialogues in terms of place and participation was considered commendable by most of the respondents. The dialogue process was principally led by the MoH’s Planning and Policy Department with technical support from the staff of the WHO country office in Liberia. The respondents revealed that the dialogue process involved identification of themes by a small group that included the WHO the MoH’s Planning and Policy Department focal person. Once the theme was agreed upon a specialist was invited to make a presentation on it:

*For instance, if they want us to focus on maternal health issues, they will have a specialist on maternal health issues come and do a presentation on best practices from other countries, then they will have the county health officers make presentations on issues from their counties to see the level at which each county is… and people will discuss the way forward and what could be done better.* (MoH official)

Once the issues and activities were agreed upon in the general meetings, smaller meetings were convened between MoH and WHO officials to agree on the practicalities of implementation. Despite there being a clear process for the dialogues, several shortfalls with the technicalities of the procedures were highlighted. Primarily it was felt that the dialogues were not structured well enough to capture and articulate all the key issues. Most respondents were of the view that the dialogue process did not follow a specified methodology that included the generation of ideas, building of consensus and agreement on the way forward based on an informed-decision process. Two of the respondents noted,

*The mix of people who participate in dialogue activities is good. But the way of generating and processing ideas is not very effective. The skills of the facilitators are not particularly at the right level.* (Donor representative)

*There are no analytical tools like the strengths, weaknesses, opportunities and threats (SWOT) framework used to analyse issues to come up with solutions. In my opinion, there were no critical discussions around issues because there were no defined methodologies for the deliberations. Further, I am not sure that the proper outcomes were generated.* (Donor representative)

The management of presentations was considered to have been improper in terms of how time was handled and how the main points were generated. The result was superficial discussion of the issues. The respondents believed that little attention was given to preparing and distributing background information or documents ahead of time, which might have contributed to making the discussions more informed. One of the respondents lamented,

*You cannot come to a conference and be expected to read 50 pages given to you a day before the meeting and give proper input.* (NGO national level representative)

Lack of continuity among the meetings was raised as a concern by the respondents. Despite the time spent in the dialogues, there were instances where some of the suggested activities were stopped or were not implemented owing to competing priorities. There were respondents who felt that the dialogues were not different from the regular meetings, that they were more of a ticking-the-box exercise or another form of process to ensure that the counties were accountable for implementing their plans, as one of them explained,

*It is just another checklist for the counties, because they don’t show that the problems have been overcome with the resources provided for them. I think one of the issues the counties recognise from this has been that it is mainly a means of monitoring them. It’s a good way to show in a transparent way and hold the counties accountable for what they plan to do. But it’s also a means of shaming the counties. If the counties make plans but do not achieve them, everyone will know that, but they will not understand that they couldn’t be achieved without an enabling environment and resources to move from one point to another.* (MoH Sub-national representative)

### How the EVD outbreak influenced the policy dialogue

The policy dialogues took a unique shape during the Ebola outbreak. During the early days of the outbreak, the MoH was rendered powerless, as the leading role in dealing with the disease was taken over by politicians and donor groups. Some respondents even attributed the poor response and delay in inhibiting the outbreak to the muddled leadership. The respondents believed that once the ministry had reassumed its role, it managed to control the situation:

*The Ministry of Health can also take consolation in the fact that the response to Ebola was taken over by politicians. They even skewed the initial response of the public. Some accused the ministry of health of raising a false alarm about Ebola in order to get extra money*. (Sub-national NGO representative)

From the study, it was generally felt that the processes of the dialogues were better organised during the Ebola outbreak. After the initial confusion, the returning of the coordination role to the MoH made the ministry more authoritative and responsive. The ministry went the extra mile by assigning responsibilities and coordinating the partners, as one of the interviewees explained,

*The ministry came up with the issues and presented them to the donors, saying, “partners, this is what is required, and what can you offer?” They even went further to streamline activities among the different donors/partners. For example they would say, “This organisation is taking the lead in safe and dignified burial during the Ebola crisis, so you can tackle another area.”* (NGO national representative)

In relation to the dialogues both before and during the Ebola outbreak, it was perceived that some of the dialogue documents had been produced in a hasty manner, which potentially affected the quality and inclusiveness of the dialogues, as one of the respondents stated,

*I think there is tension that exists between the external demands to produce a product by a certain deadline…which might affect the content and quality*. (Sub-national level representative)

From a procedural perspective, the process of developing the investment plan was cited in many instances as having been carried out in a rush with inadequate consultation of Liberians. This is what two respondents said about it,

*Very recently, with a number of technical experts from outside, we tried to develop the investment plan for Liberia to restore itself after the Ebola crisis. My impression of it was that it went so fast that a lot of people did not sort of know what was going on. This was by necessity not because the intention was to be non-transparent, but it just had to go quickly because there were funding deadlines that had international implications and could not be shifted. My impression was that there was a plan that was produced, but largely, except for the people who were deeply involved in it, people weren’t really appropriately familiarised with the plan afterwards.* (National level representative)

… *it’s great to have this health investment plan and it’s proved to be a wonderful fund-raising tool for the country, for the president, for the minister, but it’s certainly, I think, something that people are still sort of, like, “What’s actually in there?” “Who put that in there?” “Why is that in there?” To me, I just see that as sort of like a victory in one … But it doesn’t seem to be owned by even the middle management and other ministry stakeholders*. (National level NGO representative)

### Priority issues and supportive evidence discussed during the dialogues

There was general consensus among the respondents that the dialogues were addressing pertinent issues of the Liberian health system. The priority issues included the health system’s performance, health financing, human resources for health, and emergent health situations such as the Ebola outbreak (see Table 3).

**Table 3:** Issues featured in policy dialogue meetings in Liberia

Area	Specific issues
Health systems performance	• Analysis of the past period compared with targets • Health facility accreditation process
Health financing	• Resource mapping – of either allocated resources or commitments from partners within the sector • Investment plan and health financing and its sustainability
Other health systems issues	• Supply chain management • Service delivery and its infrastructure improvement • Monitoring and evaluation, monitoring and evaluation framework, research, and some activities under health information systems • Drugs and supplies
Human resources for health	• Health workers’ employment and salaries • Health workers’ satisfaction and motivation • Staff attrition, retention and attraction to rural areas
Emergent situations	• Ebola outbreak • Measles outbreak • Health and hygiene promotion programme • Surveillance • Incident management system • Infection control

Opinions about the process for generating and selecting priority issues for the dialogues were extremely divergent among the respondents. A number of them were not aware of how the themes were generated or decided upon. A few others who were knowledgeable of that process described it as having evolved from several proposals at the MoH from which at least three priorities were selected and circulated to the main stakeholders such as the United Nations Population Fund, WHO, the United Nations Children’s Fund and the United States Agency for International Development, and all the other health partners for the final decision. The respondents felt that only a few of the health partners had an important role in the decision on the priorities.

Sometimes the issues for discussion were generated from various departments within the MoH, such as in instances where an issue could not be resolved in the department. For example, the decisions to generate evidence or collect data on user fees and to conduct perception studies on the health systems were all justified by departmental needs.

Some respondents felt that the planning systems had been structured in a way that allowed the policy issues discussed in the dialogues to be determined by external forces such as the global health initiatives, international donors, and partners, often biased by their interests. As a result most of the policy issues given priority were predetermined and some evolving issues in the health sector were not accorded prominence. The respondents also perceived certain issues to have been driven by the funders of the dialogues, such as those cited in these examples,

*Our biggest challenge is the fact that people want to have certain things done, for example issues related to national health accounts. How many people know what a national health account is or what it means? People don’t understand but the ministry included it, as it was influenced by donors.* (National level representative)

… *with respect to how we build consensus on this policy dialogue thematic areas, that discussion has largely been between the Ministry of Health and our technical colleagues at WHO.* (MoH coordinator, M&E)

The validity of the data used in the dialogues was a concern cited by a number of respondents, such as the one who made this comment,

*For example, somebody comes and presents wrong data about Sinoe and I know the situation, and I wonder where they got that data.* (Sub-region NGO representative)

Time was cited as a major stumbling block, as there were usually too many issues to be discussed within the allocated time for the dialogue. This led to superficial discussion of issues with the proposed solutions and interventions being suboptimal.

## Lessons learned

Several lessons were deduced from the dialogue process as experienced by the respondents. These lessons pertained to the need for financial sustenance for the dialogues, for better coordination of partners, for community engagement, for alignment of policy dialogue with political will, for resiliency in the health systems, and for quality control. Table 4 outlines the lessons and examples.

**Table 4:** Summary of lessons learned from the policy dialogue processes

Areas	Lessons
1. Flexible funds	“Well, I will always talk of the indirect effect of having the dialogue. The fact that we had mapped out partners, we knew who to talk to initially. And with resource mapping, partners’ resources that were available and could not be used for the normal health services had to be directed to Ebola. I would say that those were some of the good outcomes, though indirect, of the policy dialogue.” (National level MoH official)
	“I think critical issues that need to be addressed to improve any institution or any sector. And so if you have a grant that facilities this kind of work, I think it is a good thing because there has never been such a grant since I have been in this ministry that has supported policy-specific and related issues or issues that are not funded by major projects but are very important. I think this is a good lesson that we can learn from that.” (Senior MoH official _ national level)
2. Alignment of partners	“… We learned more about how to come together and distribute responsibilities. I think we also learned how to make the process more inclusive, like in involving the community.” (Senior MoH official _ national level)
	“Responsibilities are shared, a partner may be designated as the lead. For example, the pillar lead hygiene promotion has been UNICEF (United Nations Children’s Fund). So, they take the lead on that. And all other partners that are part of that group will follow and do the coordination.” (CSO Representative, national level)
	“The main lesson I will take from this is that coordination and good results are effective when partners have a common forum for dialogue. It has helped to give some direction to all in the health sector. What I mean is that the ministry could have prepared the policies and plans, but I don’t think that alignment with partners, which gave a common direction for all stakeholders, is possible without dialogue.” (Senior MoH official _ national level)
3. Coordination	“Finding money that we can use for a consolidated operational plan for the sector is one lesson that we can learn as well, instead of having people with their individual work plans. But you need to bring them together so that you see the entire health sector plan.” (Senior MoH official _ national level)
	“The positive lesson I have taken from this is that the MoH continued the coordination function of the health sector after Ebola. Just yesterday someone was saying that we are moving to development, not just recovery.” (CSO Representative, national level)
4. Community engagement	“… I don’t know if that will stick, but I do see that somehow people are starting to realise that without that component and without trust in the whole system, and without engagement between communities and their government, you are going to have a tough time responding.” (CSO Representative, national level)
5. Alignment of policy dialogue with political will	“Let’s start with the things that went really well. I think aligning the political will of the Presidency with the Ministry of Health with sort of key leaders was a good way to ensure at the highest level that the dialogue was consistent, whether or not what was being said in a meeting in the Ministry of Health was the same thing as what was being said in a meeting with the President.” (CSO Representative, national level)
6. Need for resilient health systems and quality control	“Another lesson learned was that our health system should be strengthened, because when some of our health facilities were built, our people were not thinking to meet present day realities. You know those places were just built because we needed health facilities; they are just tight and we need to make them elaborate so that we will have some of the services there for people to access.” (MoH official Sub national level)

## Discussion

Our study respondents had different perceptions on the intent of the policy dialogues. To some of them the dialogues were a funding programme, while to others they were a yearly meeting. The varied understanding of the purpose and role of the dialogues in itself could create wrong expectations, which if not fulfilled could cause the actors to lose interest in the process. Indeed, some of the shortcomings mentioned, such as *“…policy dialogue funding allocation is spread out thinly over many activities …”* stemmed from misunderstanding what policy dialogues intended to achieve. The variety of perceptions represents a missed opportunity to realise the benefits of policy dialogues, which have been stated as strengthened partnerships, consensual solutions and joint ownership of policy decisions [1, 3, 19].

Policy dialogues in Liberia were characterised as participatory, with a range of stakeholders involved. Their composition was based on the area of discussion. They included people who dealt with policy implementation, mid-level managers, NGO representatives, policy-makers and donors. There were concerns that some of the forums were unsuitable for extensive and free dialoguing. This was particularly in regard to the dialogues that were conducted in meetings with large audiences, those with group work that was somehow dominated by a few talkative participants, and those that followed usual meeting procedures involving several presentations. Such practices are contrary to the recommendation that policy dialogues should involve small groups with enough time to debate and understand the main issues [20].

One positive aspect was the involvement of sub-national actors as part of the initial thinking and discussions in the policy-making process. These were important constituents of the policy dialogues, as literature shows that policy-making does not end with the production of policies but continues to their implementation [21]. Several factors influence how frontline implementers operationalise a policy, but often these factors are not considered during policy-making and decision-making processes. As a result policy implementers intentionally or unintentionally reshape the policies to accommodate the factors [5, 22–24]. On the negative side, the effective engagement of sub-national health managers was hampered by poor facilitation, lack of skills in policy dialogue and use of inappropriate methodology for the dialogues. Some of sub-national health managers were intimidated when required to respond to questions on their presentations in the meetings.

There has been a push for community participation in the policy process and service delivery as a means of accommodating people’s wishes [25–27]. However, just including community level participants in policy-making activities is not enough to engender effective policy-making. In the Liberian policy dialogues, community participation varied, but there was no clear definition of who should be involved. The lack of well-established community structures is a documented hindrance to realising community participation [26, 28].

Policy dialogue processes need to be collaborative, providing room for interactive knowledge sharing and continued learning [29, 30]. Such a process accords the opportunity for actors to learn from each other, which enhances interactive participation, harmonisation of ideas and approaches, and consideration of the implementation process [31]. Realising this objective calls for consistency in participation as well as skills to participate in interactive knowledge exchange and learning. A shortcoming highlighted in our study was that in some cases the individuals delegated as participants to the dialogues were not of the expected skill or knowledge level.

The literature stresses the importance of conducting good policy dialogues [1, 31, 32]. But to ensure their quality means surmounting weaknesses such as those highlighted in our study, which were the late distribution of documents, the use of inappropriate methods, and the use of data of doubtable quality. The manner in which documents are distributed should contribute to effective information sharing and communication, which requires the use innovative and multiple approaches [33, 34]. For example, evidence and policy documents may be shared through forums, newspaper columns and other mass media outlets, informal conversations, online social media, legislative hearings and lobbying [31]. The late distribution of bulky documents during the Liberian dialogues may have partly been responsible for what the respondents referred to as superficial discussions and sub-optimal decisions.

It is evident from our study that the issues addressed in the policy dialogues were relevant to the Liberian health system, especially those pertaining to the improvement of alignment of actors’ actions and investments, improvement of community engagement and building of health systems’ resilience. All of these had been identified as challenges in Liberia [13, 17, 35]. Policy dialogues need to focus on issues relevant to the community in order to be legitimate [3, 32].

The authenticity of the policy dialogues in our study was compromised by several processes that were deemed not to be transparent. Firstly, the themes of the policy dialogues were thought to have been identified by MoH and WHO with little consultation with the other stakeholders. Proper methodology is required for raising the issues, for the debating procedures and for decision-making for the policy dialogue process to be strong. Competent facilitation to guide the dialogue process is equally essential [2, 3, 20]. The respondents in our study noted that the lack of a systematic approach in the dialogues compromised participation of stakeholders *“… there are no analytical tools like the strengths, weaknesses, opportunities and threats (SWOT) framework used to analyse issues to come up with solutions …”*

Secondly, some of the information presented during the dialogues was considered not to be accurate. Such perceptions could affect the trust and buy-in of stakeholders and eventual implementation of the policies. We emphasise that evidence be used to inform the policy process and that the evidence be owned by the stakeholders in the policy dialogue [25, 36–38]. This is even more important in situations where the policy issue under consideration is contested [9, 39], in which case the evidence must of good quality to be trusted [40]. The limited use of evidence as noted in our study may be attributed to the perceived poor quality of available data.

Our study found that there was weak follow-up on agreed actions and constraints in carrying out all the activities planned during the policy dialogue owing to lack of funding. Furthermore, lack of consistency in participation in the dialogues was reported. Active and sustained participation in the dialogues is key for the actors to learn from the different perspectives of others. The literature highlights the need for adequate resource commitments in terms of time, expertise and funding in order to have effective, successful and participatory policy dialogues [8, 41, 42]. In addition, constant discussions and negotiations are necessary and continual work is required to maintain cohesive and unified commitment to the established agenda [36, 43, 44]. In institutionalising policy dialogues, these have to be ensured.

Our study supports the notion that the contexts under which policy dialogues occur are important. Context has an influence on the stakeholders’ participation, contribution, collaboration and decisions [8, 42]. In our study the influence of two contexts stood out clearly. One context was the hierarchical nature of the health systems, compounded by donor dependency, which was responsible for their excessive influence in the whole policy dialogue process. The MoH was regarded as an important actor in the policy dialogues and as ultimately responsible for decision-making. However, there was disjuncture in the process, as the decisions on the policies and activities were dependent on funding from donors. In several instances, the respondents lamented about the funding limitations for post-dialogue activities.

The second context was the Ebola outbreak, during which the role of the MoH was taken over by politicians, rendering the ministry powerless to address the critical health issues surrounding the outbreak. Once the MoH went back into its role, it adopted a revolutionary approach to the crisis that was not its usual way of doing business: it concentrated on practical issues and involvement of the community in the real sense to get their buy-in.

## Study limitations

Caution should be exercised in generalising these results given the unique context of Liberia. Results are generalizable in as much as the context is the same. This withstanding, we believe that our study provides lessons that can improve policy dialogue processes in low income countries.

## Conclusions

Health policy dialogue has created a platform for policy discussions and decisions in Liberia. It is a process that is well recognised and appreciated and is credited for the success of the negotiations during the Ebola outbreak in Liberia. Borrowing from the policy dialogue programme of the Australian Agency for International Development (AusAID), and its associated literature [36, 45] and learning from our study, we cement the argument that important elements for effective policy dialogues include the extent to which it is clear what is to be achieved through the dialogue, or clarity of intent; the balance of power, knowledge and ownership, or negotiating capital; the capabilities and characteristics of the actors or individuals involved; the forum used for the dialogue, which are the formal and informal spaces and opportunities to understand each other’s values and interests; and the evidence, or the extent to which data and analysis inform the dialogue, and who owns the data [36, 45]. The context in which policy dialogue occurs, will influence these elements [36, 45].

Policy dialogue processes need to be transparent right from the selection of themes for dialogue and systematic approach to the dialogue instituted. Relevant capacity needs to be built at the different levels. Sub national actors should be empowered with training on presentation skills so as to confidently present and defend their information and what they perceive as important. Their active participation will contribute to effective follow-up and implementation of policies. In order to sustain and strengthen the policy dialogues there needs to be proper information sharing through various forums, facilitation competence, and use of good quality evidence to ensure that the process is credible and legitimate.

## Abbreviations

AusAID, Australian Agency for International Development; M&E, monitoring and evaluation; MoH, ministry of health; NGO, nongovernmental organisation; WHO, World Health Organization.
